# Supplementary material for: RKIP Regulates Differentiation-Related Features in Melanocytic Cells
Source: Cancers (Basel). 2020 Jun 3;12(6):1451. doi: 10.3390/cancers12061451 (PMC7352799; doi:10.3390/cancers12061451)
Supplement: Supplementary file 1 [file cancers-12-01451-s001.pdf]

Supplementary Materials: RKIP Regulates Differentiation-Related Features in Melanocytic Cells

Cristina Penas, Aintzane Apraiz, Iraia Muñoa, Yoana Arroyo-Berdugo, Javier Rasero, Pilar A. Ezkurra, Veronica Velasco, Nerea Subiran, Anja K. Bosserhoff, Santos Alonso, Aintzane Asumendi and Maria D. Boyano

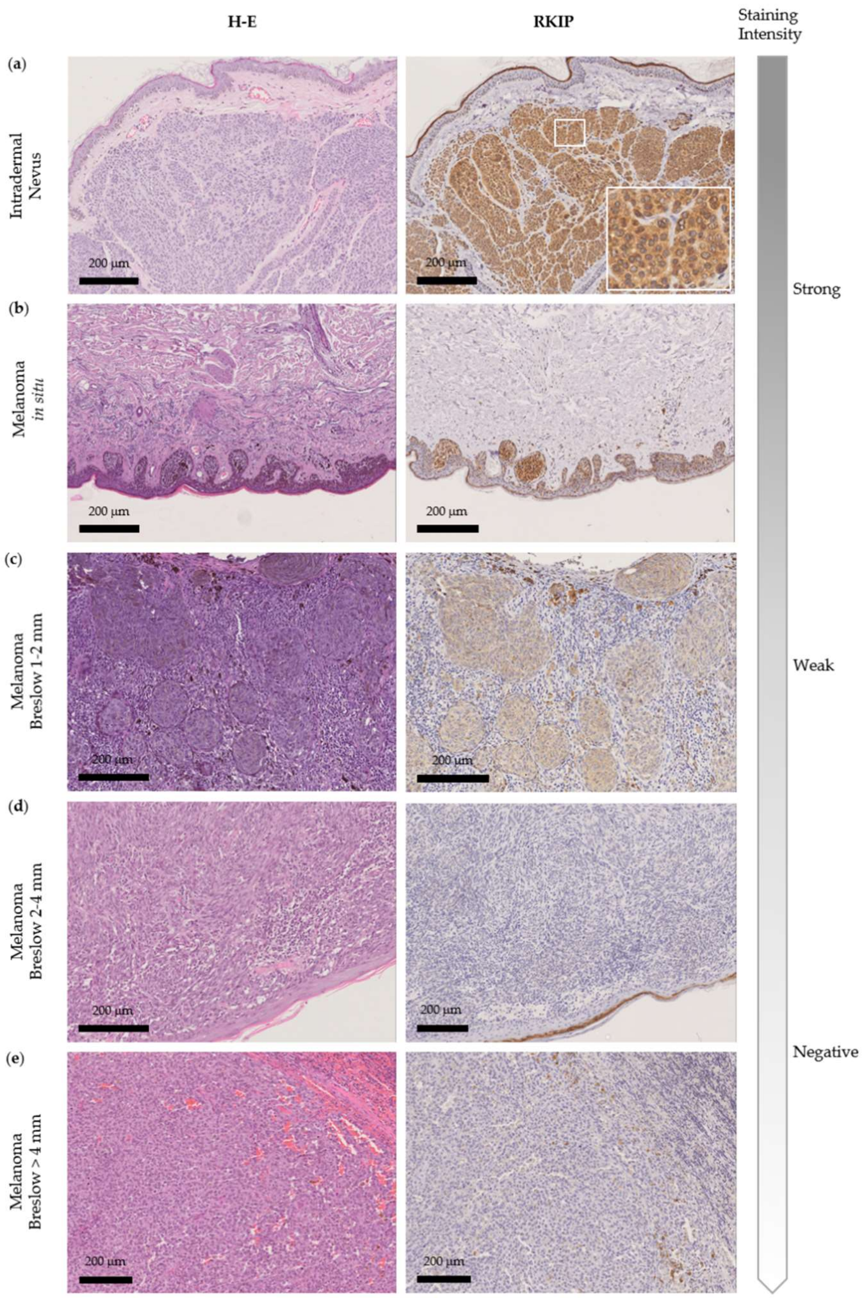

Figure S1. Representative images of manually scored RKIP staining categories in FFPE biopsies from patients. At the feft: Hematoxylin-Eosin staining (H-E). At the right: Immunohistochemistry (IHC) of RKIP for increasing Breslow Index. (**a**-**b**) Strong staining for intradermal nevus and in situ melanoma, respectively; (**c**) weak staining for a melanoma with Breslow Index between 1 and 2 mm; (**d**-**e**) negative staining for melanomas thicker than 2 mm.

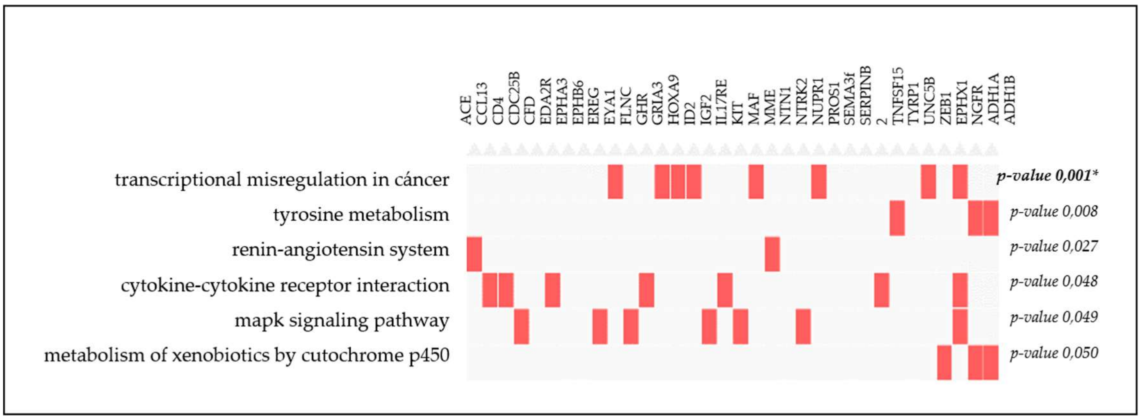

Figure S2. RNA Sequencing data analysis. Each row of the figure represents an over-represented gene signature generated by analyzing the up-regulated and down-regulated genes from a differential expression analysis of RKIP Knockdown melanocytes HEMn-LP. Results from the EnrichR Website with a *p*-value > 0.05.

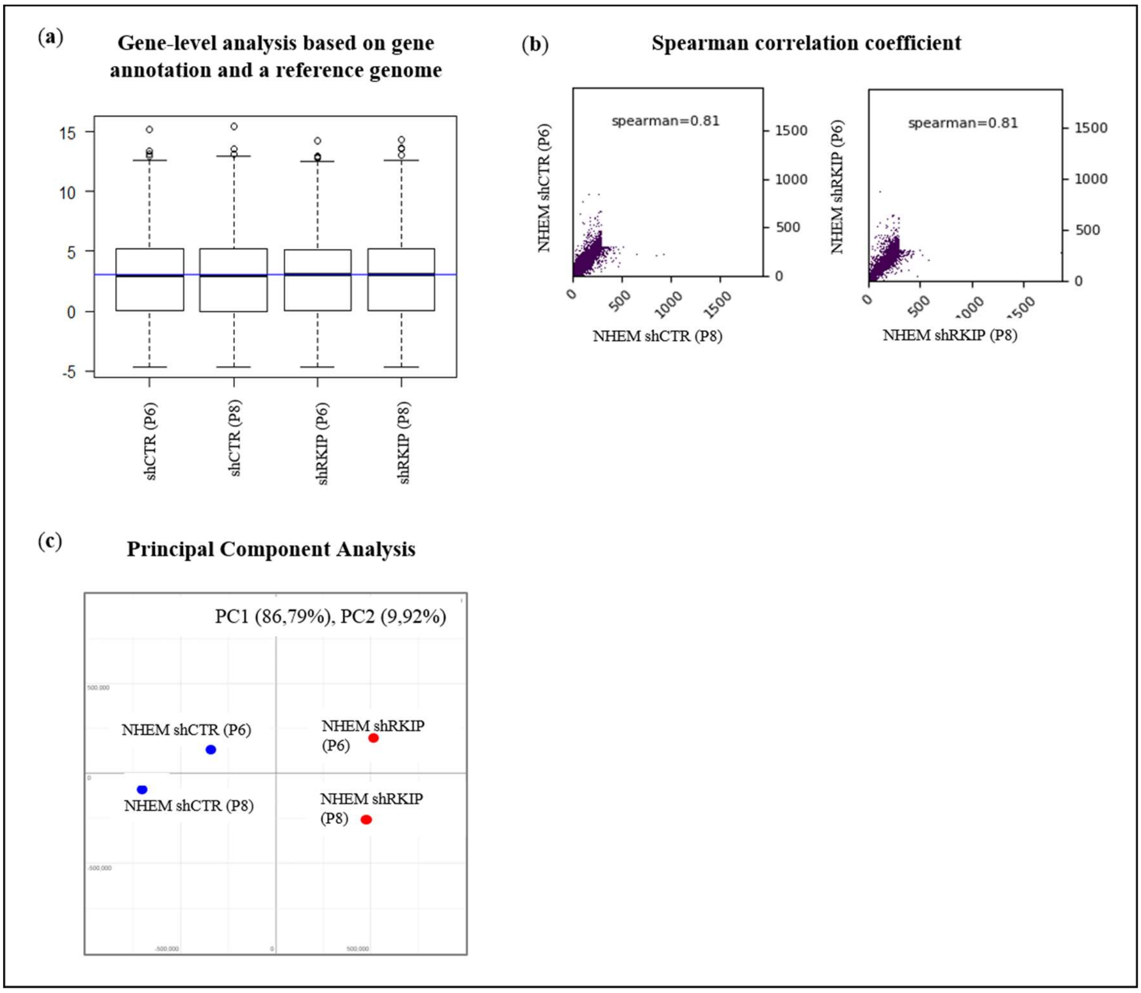

Figure S3. RNA Sequencing data quality. (**a**) Read counts normalization per sample; (**b**) Spearman correlation coefficient between replicates; (**c**) Principal component analysis for replicates.

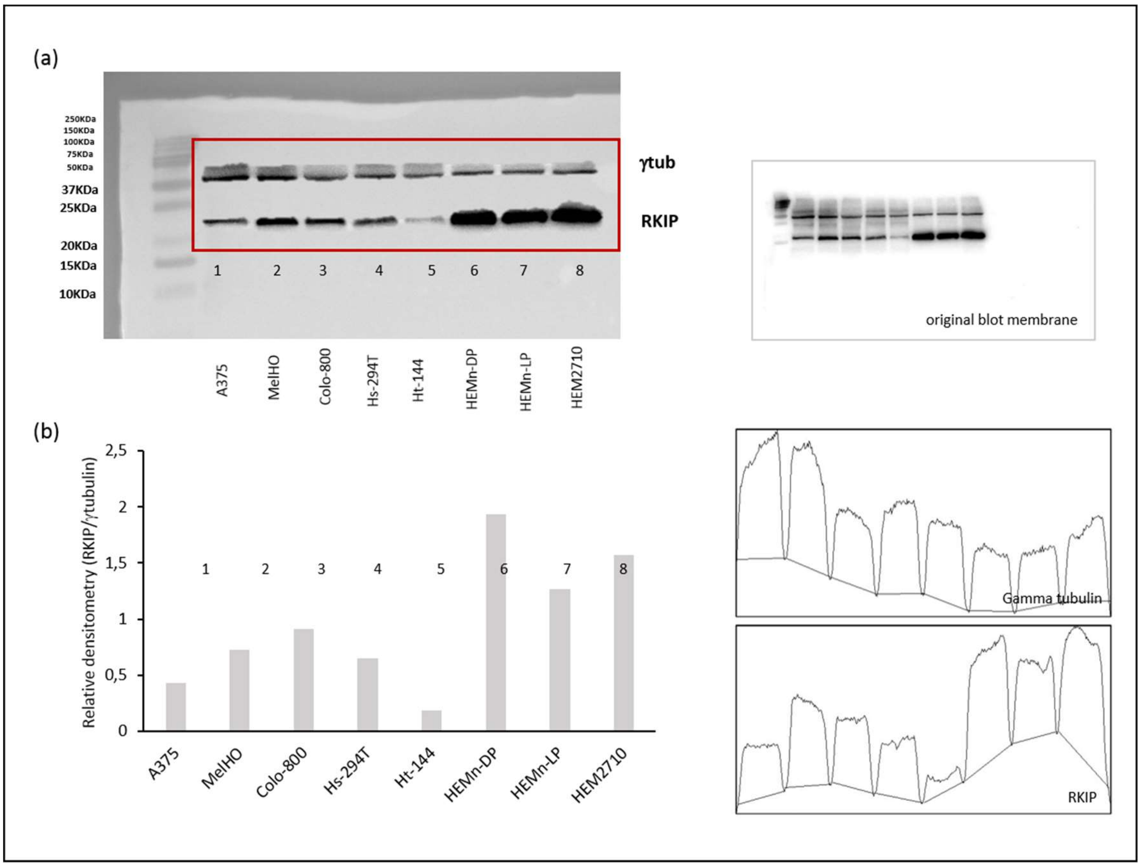

Figure S4. Details of Western Blot from Figure 2 related with RKIP expression in several cell lines. (**a**) Blots for RKIP and γTubulin in primary and metastatic melanoma and in three primary melanocytes HEMn-DP (dark pigmented), HEMn-LP (light pigmented) and HEM2710. On the right, original blot for each analyzed protein. Highlighted with a red square the information included in the Figure 3 (horizontal rotation). (**b**) Densitometry from the plots showing the relative level of RKIP/γtubulin. On the right, the peak plots for each protein analyzed (γTubulin and RKIP, respectively).

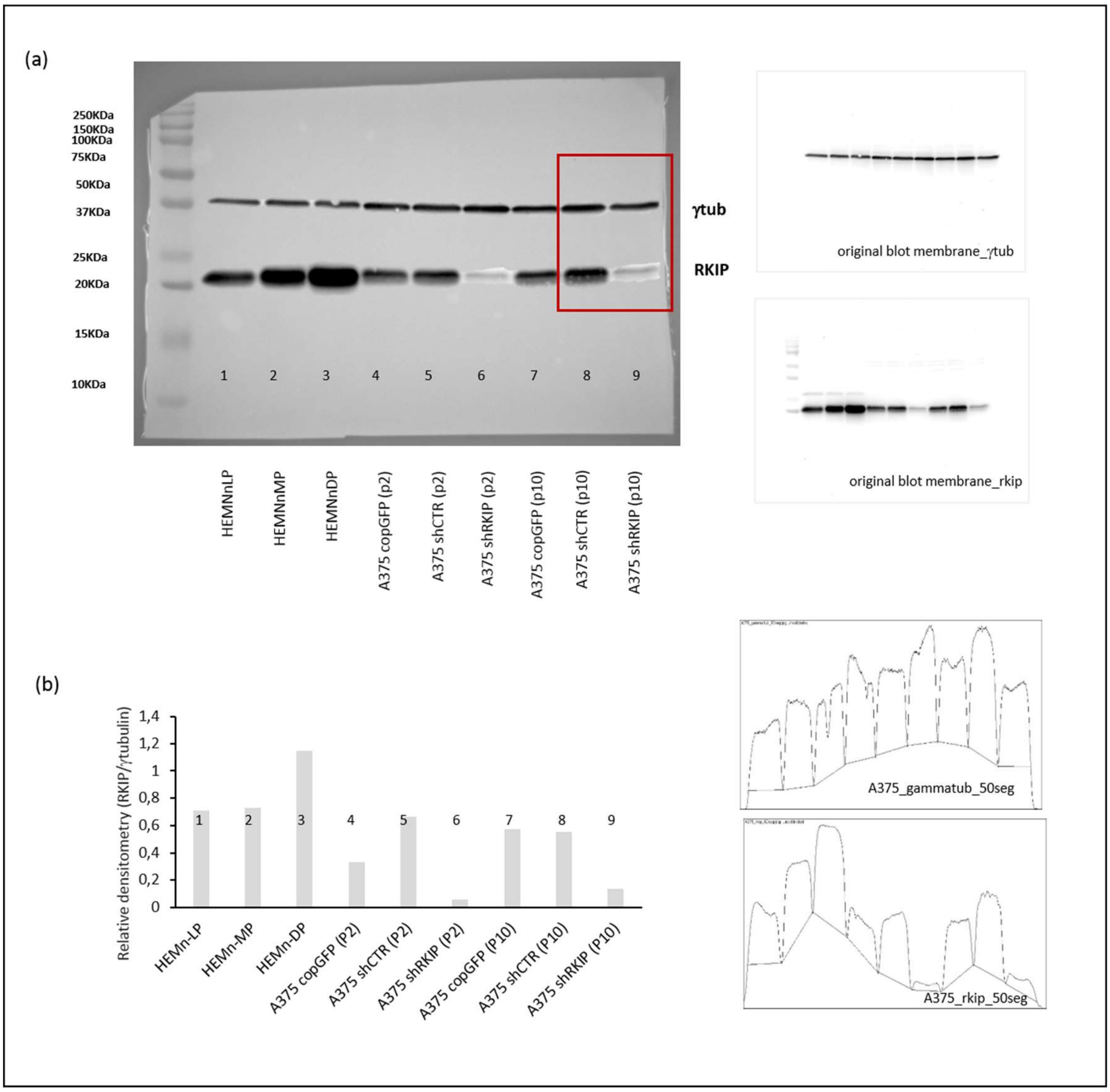

Figure S5. Details of Western Blot from Figure 3 related to information of A375 melanoma cell line. (**a**) Blots for RKIP and γTubulin in three primary melanocytes HEMn-LP (light pigmented), HEMn-MP (medium pigmented) and HEMn-DP (dark pigmented) and in A375 melanoma cells (two consecutive passages after transfection for down regulation of endogenous RKIP). On the right, original blots for each analyzed protein. Information included in the Figure 3 has been highlighted with a red square. (**b**) Densitometry from the plots showing the relative level of RKIP/γtubulin. On the right, the peak plots for each analyzed protein (γTubulin and RKIP, respectively).

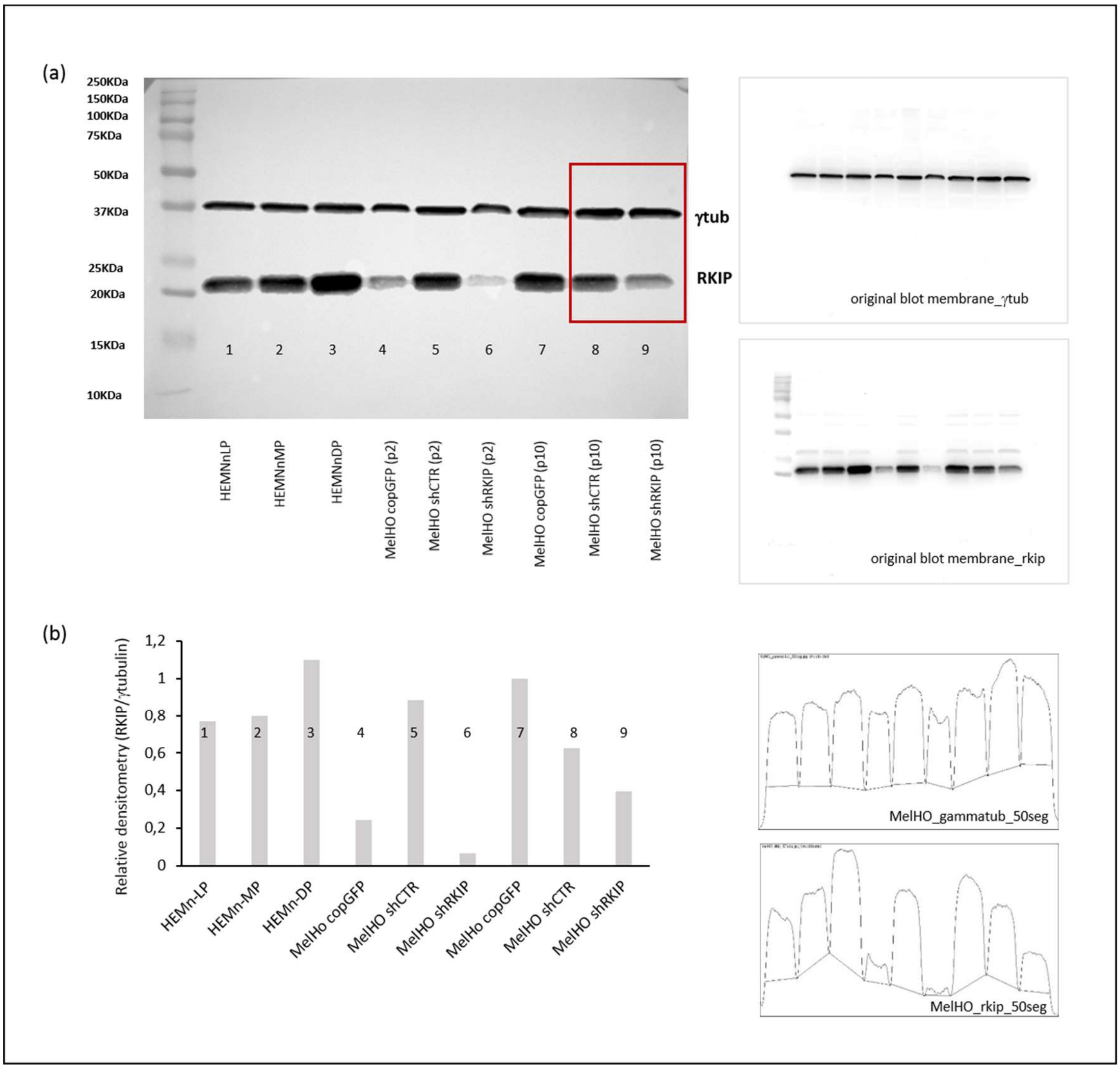

Figure S6. Details of Western Blot from Figure 3 related to information of MelHO melanoma cell line. (**a**) Blots for RKIP and γTubulin in three primary melanocytes HEMn-LP (light pigmented), HEMn-MP (medium pigmented) and HEMn-DP (dark pigmented) and in MelHO melanoma cells (two consecutive passages after transfection for RKIP down regulation). On the right, original blots for each analyzed protein. Highlighted with red square the information included in the Figure 3. (**b**) Densitometry from the plots showing the relative level of RKIP/γtubulin. On the right, the peak plots for each protein analyzed (γTubulin and RKIP, respectively).

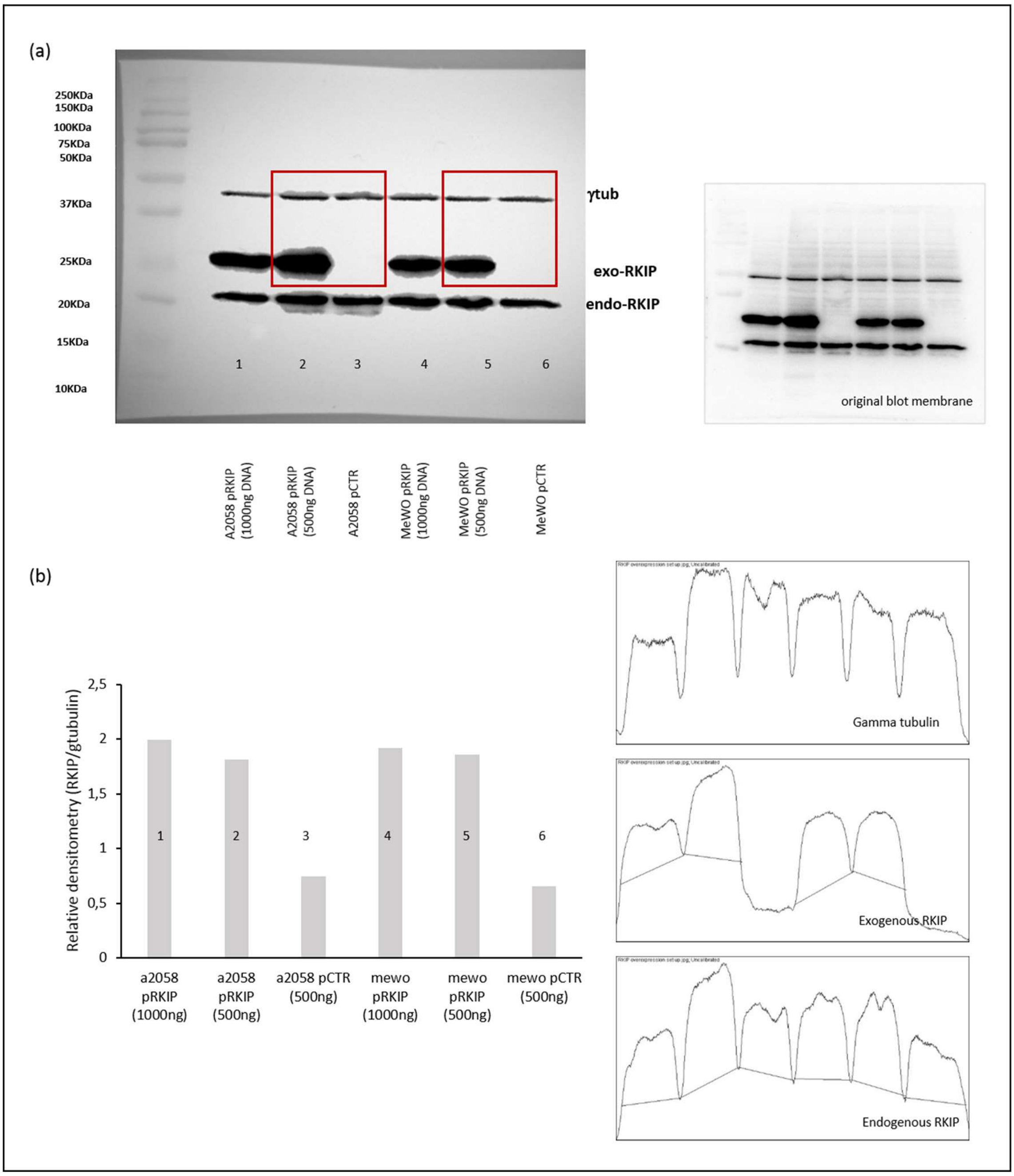

Figure S7. Details of Western Blot from Figure 4 related to the information regarding MeWO and A2058 melanoma cell lines. (**a**) Blots for RKIP (1000 ng and 500 ng of plasmid for overexpression of RKIP) and γTubulin. RKIP plasmid (Myc-DDK-tagged) encodes for a RKIP protein 4KDa bigger than the endogenous RKIP. Therefore, blots belonging to transfected samples contain 2 lines (endogenous and overexpressed forms of RKIP). On the right, the original blot. Red squares highlight the blot fraction employed for the Figure 5 (horizontal rotation). (**b**) Densitometry from the plots showing the relative level of RKIP/γtubulin. In this case, the values of endogenous and exogenous RKIP have been added to calculate relative expression. On the right, the peak plots for each analyzed protein (γTubulin, exogenous RKIP and endogenous one, respectively).

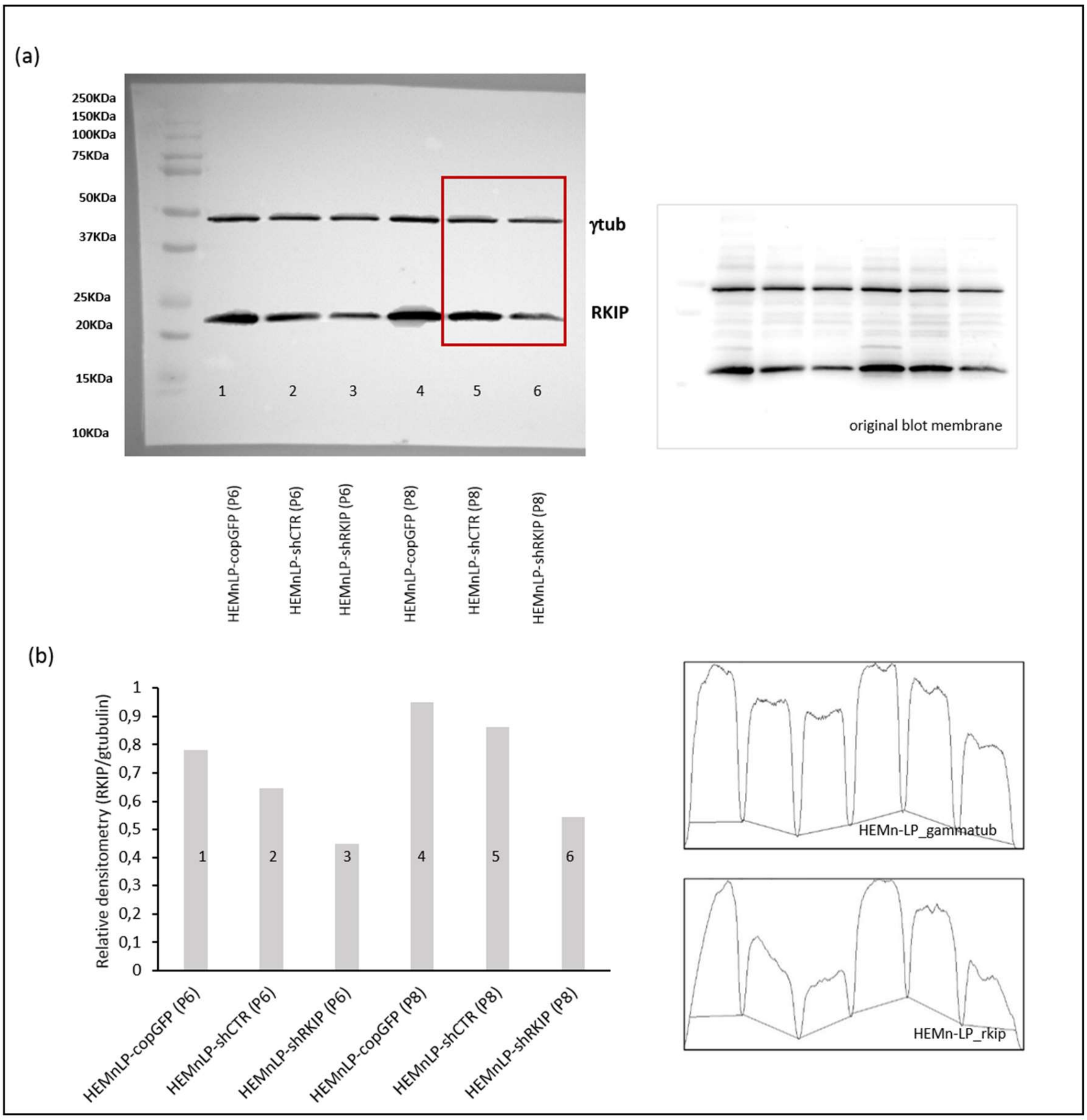

Figure S8. Details of Western Blot from Figure 5 related to primary melanocytes (HEMn-LP). (**a**) Blots for RKIP and γTubulin in two consecutive passages of primary melanocytes HEMn-LP after transfection with lentiviral particles in order to down regulate endogenous RKIP. On the right, original blot for both analyzed proteins. Highlighted with red square the information included in the Figure 5. (**b**) Densitometry from the plots showing the relative level of RKIP/γtubulin. On the right, the peak plots for each analyzed protein (γTubulin and RKIP, respectively).

Table S1. Differentially expressed genes after RKIP silencing. The details of Log Fold Change, P-Value and False Discovery Rate are specified in each column.

Gene Symbol	logFC	*p*-Value	FDR
AC008105.3	−1.935	0.001	0.049
AC025125.1	−3.192	0.000	0.006
AC025594.1	−2.906	0.000	0.024
AC079949.2	−5.287	0.000	0.015
AC115090.1	5.285	0.000	0.013
AC244153.1	5.285	0.000	0.013
ACE	−2.512	0.001	0.048
ADAMTS12	1.076	0.000	0.031
ADAMTS15	2.391	0.000	0.032
ADAP1	−1.024	0.000	0.041
ADD3	−1.340	0.001	0.048
ADH1A	2.779	0.000	0.006
ADH1B	2.784	0.000	0.014
AL359258.1	−2.534	0.000	0.038
AL390038.1	−2.551	0.000	0.038
AL512329.2	4.490	0.000	0.036
ALPL	1.145	0.000	0.041
ANLN	0.857	0.000	0.046
APOL1	−4.465	0.000	0.006
ARHGAP26	−1.144	0.000	0.038
ARHGEF28	−1.141	0.000	0.038
ARHGEF6	−1.208	0.000	0.027
ATP10A	−2.236	0.000	0.031
AURKC	−3.040	0.000	0.022
BCL3	1.156	0.000	0.024
BST2	−1.184	0.001	0.049
C1orf216	−1.123	0.000	0.033
C1orf54	−3.151	0.000	0.040
C1QTNF7	−3.849	0.000	0.038
C21orf91−OT1	−1.106	0.000	0.041
C2CD6	2.086	0.000	0.028
C8orf31	1.990	0.000	0.037
CAPG	2.620	0.000	0.022
CCDC3	2.572	0.000	0.041
CCL13	1.650	0.001	0.048
CD302	1.620	0.000	0.023
CD4	−5.673	0.000	0.017
CDC25B	1.247	0.000	0.038
CDK18	2.649	0.000	0.013
CDO1	2.221	0.000	0.024
CDON	1.526	0.000	0.024
CECR7	−0.932	0.001	0.049
CENPF	1.176	0.000	0.041
CES4A	1.919	0.000	0.041
CFD	−2.226	0.000	0.024
CHODL	−4.067	0.000	0.020
CHRDL1	3.451	0.000	0.014
CHRFAM7A	2.553	0.000	0.041
CLDN1	−2.065	0.001	0.049
CLSTN2	2.887	0.000	0.006
CNTN4	−5.823	0.000	0.010
COL22A1	−5.561	0.000	0.019

COL8A1	1.850	0.000	0.040
CPED1	−1.193	0.000	0.019
CSPG4P13	−0.995	0.000	0.040
CTSW	3.543	0.000	0.040
DIO2	1.512	0.000	0.027
EDA2R	−0.967	0.000	0.040
EEF1A2	−3.087	0.000	0.018
EFEMP1	1.972	0.000	0.014
EGLN3	2.434	0.000	0.031
EHBP1L1	−0.830	0.001	0.047
EPHA3	−6.092	0.000	0.007
EPHB6	5.366	0.000	0.016
EPHX1	−0.928	0.000	0.033
EREG	−1.663	0.000	0.027
EYA1	−2.306	0.000	0.034
FAM167A	1.166	0.000	0.031
FAM171A1	2.078	0.000	0.013
FAM180A	7.677	0.000	0.014
FAM198B	1.690	0.000	0.022
FAM198B-AS1	2.419	0.000	0.040
FAM43A	−1.285	0.000	0.042
FBXO32	0.937	0.000	0.040
FILIP1L	−1.272	0.000	0.023
FLNC	−2.706	0.000	0.006
FMNL1	−1.727	0.000	0.023
FMO6P	2.394	0.000	0.031
FRG1BP	−1.537	0.000	0.041
FRG1CP	−0.831	0.001	0.049
FRMD3	2.286	0.000	0.021
FRZB	−2.818	0.000	0.008
GALNT16	−1.693	0.000	0.031
GHR	−2.038	0.000	0.025
GLB1L	−1.573	0.000	0.014
GLP2R	5.737	0.000	0.041
GPR143	−5.630	0.000	0.014
GPRC5A	−0.951	0.001	0.049
GREB1L	1.464	0.000	0.038
GRIA3	3.504	0.000	0.006
GRID1	−7.459	0.000	0.014
H19	3.804	0.000	0.023
HAGLR	−3.058	0.000	0.028
HCAR1	3.204	0.000	0.019
HDAC9	−1.629	0.000	0.032
HOXA9	1.258	0.000	0.040
HOXB13	−9.156	0.000	0.007
HOXD9	−4.882	0.000	0.038
HS3ST3A1	2.829	0.000	0.020
HS3ST3B1	2.865	0.000	0.006
HSPB6	−3.068	0.000	0.041
HSPB7	−4.167	0.000	0.018
ID2	1.884	0.000	0.023
IGDCC4	−1.672	0.000	0.041
IGF2	3.920	0.000	0.016
IGFN1	6.423	0.000	0.014
IL17RE	2.597	0.000	0.036
IRF4	−4.794	0.000	0.023

KCNA3	−4.644	0.000	0.032
KCNK2	3.263	0.001	0.049
KIAA1217	−7.795	0.000	0.006
KIAA1324L	3.482	0.000	0.013
KIAA1549	1.842	0.000	0.034
KIAA1755	−1.303	0.000	0.041
KIF20A	1.127	0.000	0.040
KIT	1.170	0.000	0.024
KLF4	−1.204	0.000	0.028
L3MBTL4	−2.143	0.000	0.028
LANCL3	3.901	0.000	0.023
LINC00987	2.001	0.000	0.042
LINC01597	−6.389	0.000	0.035
LINGO1	−1.950	0.000	0.028
LRP5	−1.804	0.000	0.032
LRRC32	2.404	0.000	0.014
LRRN3	−1.662	0.000	0.022
LYPD6B	1.944	0.000	0.038
LZTS1	0.911	0.001	0.049
MAF	2.860	0.000	0.013
MAL	−4.804	0.000	0.023
MCHR1	2.283	0.000	0.024
MCOLN3	−3.064	0.000	0.014
MCTP2	−6.413	0.000	0.035
MEG3	3.478	0.000	0.014
MEG9	3.475	0.000	0.018
MFAP4	2.901	0.000	0.014
MGP	2.503	0.000	0.038
MID2	−1.467	0.000	0.044
MKI67	0.842	0.000	0.041
MLANA	−4.031	0.000	0.023
MME	0.926	0.000	0.032
MOK	1.034	0.000	0.045
MS4A6E	−2.580	0.000	0.007
MTSS1	−3.194	0.000	0.040
MT-TS1	0.892	0.000	0.042
MYCL	2.101	0.001	0.048
NAP1L3	−3.186	0.000	0.032
NAV3	−1.016	0.000	0.046
NBPF22P	−2.582	0.000	0.042
NBPF4	−2.891	0.000	0.033
NCEH1	−0.994	0.000	0.038
NDRG4	−1.637	0.000	0.041
NGFR	−4.556	0.000	0.038
NOSIP	1.342	0.000	0.022
NPTX2	−7.594	0.000	0.006
NTN1	−3.269	0.000	0.023
NTRK2	1.311	0.000	0.038
NUPR1	2.203	0.000	0.014
NXPH4	2.261	0.000	0.027
OLFM1	3.534	0.000	0.018
OLFML2B	1.869	0.000	0.014
OLFML3	−1.520	0.000	0.040
OR1E1	−8.410	0.001	0.048
PAX9	−6.157	0.000	0.032
PAXIP1-AS2	−1.964	0.000	0.022

PCBP3	1.488	0.000	0.024
PCDH17	−5.334	0.000	0.006
PDE1C	1.617	0.000	0.014
PEAR1	−4.338	0.000	0.006
PID1	−2.732	0.000	0.025
PIM1	1.074	0.001	0.049
PIP	8.716	0.000	0.040
PMEL	−0.962	0.000	0.040
PODNL1	2.274	0.000	0.023
PPP1R1C	−4.739	0.000	0.038
PRELP	1.823	0.000	0.033
PROS1	−0.843	0.000	0.045
PTGDS	−4.541	0.000	0.012
PTGFRN	2.563	0.000	0.018
PTGS1	5.011	0.000	0.006
PTP4A3	2.422	0.000	0.016
PTPRD	−4.112	0.000	0.034
RAP2A	0.884	0.000	0.039
RARB	−2.438	0.000	0.019
RARRES2	1.863	0.000	0.029
RCAN2	−1.365	0.000	0.034
S100B	−3.243	0.000	0.027
SEMA3F	1.252	0.000	0.034
SERPINB2	−2.816	0.000	0.034
SHANK2	1.726	0.000	0.038
SHISAL1	2.629	0.000	0.024
SIX2	1.927	0.000	0.032
SLC15A3	−6.289	0.000	0.012
SLC1A3	1.194	0.000	0.034
SLC38A5	3.235	0.000	0.016
SLCO4A1	1.574	0.000	0.018
SLFN11	−1.501	0.000	0.018
SNTB1	−0.834	0.000	0.042
SOBP	1.792	0.000	0.037
SOD3	−1.719	0.000	0.040
SORCS1	−2.492	0.000	0.040
SOX11	4.833	0.000	0.028
ST8SIA2	−0.834	0.000	0.045
STAMBPL1	0.946	0.000	0.041
STEAP4	2.175	0.000	0.006
SYNE2	1.476	0.000	0.028
TAC1	4.640	0.000	0.013
TDRD9	−3.793	0.000	0.012
TEX11	−2.812	0.000	0.023
THBS2	1.280	0.001	0.049
THY1	4.169	0.000	0.006
TLE2	1.298	0.000	0.042
TM4SF18	−5.520	0.000	0.034
TMEM132D	5.580	0.000	0.014
TMEM200C	−2.842	0.000	0.038
TMEM204	1.071	0.000	0.034
TNFSF15	−1.394	0.000	0.022
TP53I11	1.271	0.000	0.037
TYRP1	−2.472	0.000	0.008
UNC5B	3.350	0.000	0.006
VSTM4	1.824	0.000	0.027

ZBED6CL	2.645	0.000	0.040
ZEB1	1.143	0.001	0.049
ZNF558	−4.988	0.000	0.006
ZNF560	3.943	0.000	0.032

Table S2. Enriched processes after RKIP silencing, specifying the False Discovery Rate for assignment and the list of genes for each one.

TERM DESCRIPTION	FDR	MATCHING PROTEINS IN YOUR NETWORK (LABELS)
REGULATION OF BIOLOGICAL PROCESS	0.0479	ACE,ADAMTS12,ADAP1,ANLN,ARHGAP26,ARHGEF28,ARHGEF6,ATP10A,AURKC,BCL3,BST2,CAPG,CCDC3,CCL13,CD302,CD4,CDC25B,CDON,CENPF,CFD,CHODL,CHRDL1,CHRFAM7A,CLDN1,CLSTN2,CNTN4,COL8A1,CTCFL,DIO2,EDA2R,EEF1A2,EFEMP1,EGLN3,EPHA3,EPHB6,EREG,EYA1,FBXO32,FMNL1,FRZB,GHR,GLP2R,GPR143,GPRC5A,GRIA3,GRID1,HCAR1,HDAC9,HOXA9,HOXB13,HOXD9,HSPB6,HSPB7,ID2,IGF2,IL17RE,IRF4,KCNA3,KCNK2,KIAA1549,KIF20A,KIT,KLF4,L3MBTL4,LANCL3,LINGO1,LRP5,LRRC32,LRRN3,LYPD6B,LZTS1,MAF,MAL,MCHR1,MCTP2,MFAP4,MGP,MID2,MKI67,MOK,MTSS1,MYCL,NAV3,NCEH1,NDRG4,NGFR,NOSIP,NTN1,NTRK2,NUPR1,NXPH4,OLFM1,OR1E1,PAX9,PCBP3,PCDH17,PDE1C,PEAR1,PID1,PIM1,PIP,PODNL1,PPP1R1C,PROS1,PTGDS,PTP4A3,PTPRD,RAP2A,RARB,RARRES2,RCAN2,S100B,SEMA3F,SERPINB2,SHANK2,SIX2,SLC1A3,SLFN11,SORCS1,SOX11,SYNE2,TAC1,TDRD9,TEX11,THBS2,THY1,TLE2,TMEM132D,TMEM204,TNFSF15,TP53I11,TYRP1,UNC5B,ZEB1,ZNF558,ZNF560
DEVELOPMENTAL AND DIFFERENTIATION PROCES	**0.0045**	ACE,ALPL,ANLN,ARHGAP26,ARHGEF28,AURKC,BCL3,BST2,CD4,CDC25B,CDO1,CDON,CENPF,CHODL,CHRDL1,CLDN1,CNTN4,COL8A1,EDA2R,EFEMP1,EPHA3,EREG,EYA1,FLNC,FRZB,GREB1L,HDAC9,HOXA9,HOXB13,HOXD9,HSPB7,ID2,IGF2,IRF4,KCNK2,KIAA1217,KIT,KLF4,LINGO1,LRP5,MAF,MAL,MCOLN3,MCTP2,MGP,MME,MTSS1,MYCL,NDRG4,NGFR,NOSIP,NTN1,NTRK2,NUPR1,OLFM1,OLFML3,PAX9,PCDH17,PID1,PIM1,PRELP,PTPRD,RAP2A,RARB,RARRES2,S100B,SEMA3F,SHANK2,SIX2,SLC1A3,SOBP,SOX11,ST8SIA2,STEAP4,SYNE2,TDRD9,TEX11,THY1,TLE2,TMEM204,TYRP1,UNC5B,ZEB1
CELL SURFACE RECEPTOR SIGNALING PATHWAY	0.0451	ADAP1,ARHGEF28,BST2,CCL13,CD4,CDON,CHRDL1,EDA2R,EFEMP1,EPHA3,EPHB6,EREG,FRZB,GHR,GLP2R,GRIA3,GRID1,IGF2,IL17RE,IRF4,KIT,KLF4,LRP5,MCHR1,MTSS1,NGFR,NTRK2,PIM1,PODNL1,PTP4A3,PTPRD,SEMA3F,SERPINB2,THY1,TLE2,TNFSF15,UNC5B,ZEB1
REGULATION OF CELL POPULATION PROLIFERATION	0.0295	ACE,CD4,CDC25B,CDON,EGLN3,EREG,EYA1,FRZB,GLP2R,ID2,IGF2,KCNK2,KIT,KLF4,LRP5,LRRC32,MTSS1,NDRG4,NGFR,NTN1,NTRK2,NUPR1,PID1,PIM1,RARB,S100B,SIX2,SOX11,TAC1,TP53I11,ZEB1
CELLULAR RESPONSE TO CYTOKINE STIMULUS	0.0443	ADAMTS12,BST2,CCL13,CD4,CLDN1,EDA2R,EREG,GHR,IL17RE,IRF4,KIT,KLF4,MME,NGFR,PID1,PIM1,PODNL1,PTP4A3,SERPINB2,TNFSF15,ZEB1
BEHAVIOR	0.0183	GRID1,HOXD9,ID2,KCNK2,KIT,MCHR1,MCOLN3,NDRG4,NPTX2,NTRK2,PCDH17,S100B,SHANK2,SLC1A3,SOBP,TAC1
REGULATION OF PEPTIDYL-TYROSINE PHOSPHORYLATION	0.0443	ACE,CD4,EREG,GHR,GPRC5A,IGF2,KIT,NTRK2,THY1
DEVELOPMENTAL PIGMENTATION	0.0401	GPR143,KIT,PMEL,TYRP1, MLANA
POSITIVE REGULATION OF INTERLEUKIN-10 BIOSYNTHETIC PROCESS	0.0331	BCL3,IRF4

Table S3. Genes related with development process, which are potentially targets of NANOG transcription factor.

TERM DESCRIPTION	FDR	MATCHING PROTEINS IN YOUR NETWORK (LABELS)
Genes targeted by NANOG	4.21×10^{-9}	ALPL,ANLN,ARHGAP26,AURKC,BCL3,BST2,CD4,CENPF,CLDN1,CNTN4,COL8A1,EDA2R,EPHA3,EREG,EYA1,FRZB,GREB1L,HOXA9,HOXB13,HSPB7,ID2,IGF2,UNC5B,KCNK2,KIAA1217,KIT,KLF4,MAL,MCOLN3,MME,MTSS1,NDRG4,NGFR,NOSIP,NTN1,NTRK2,NUPR1,OLFM1,OLFML3,TLE2,THY1,TEX11,PAX9,PCDH17,PID1,PIM1,PRELP,PTPRD,RAP2A,RARB,SEMA3F,SHANK2,SLC1A3,SOBP,SOX11,ST8SIA2,STEAP4,SYNE2,TDRD9

© 2020 by the authors. Licensee MDPI, Basel, Switzerland. This article is an open access article distributed under the terms and conditions of the Creative Commons Attribution (CC BY) license (http://creativecommons.org/licenses/by/4.0/).
